# Knowledge, Attitude and Practices Related to Oral Health Among Nursing Students in Davangere City: A Cross-Sectional Survey

**DOI:** 10.3290/j.ohpd.a43367

**Published:** 2020-07-04

**Authors:** Puja C Yavagal, Tanushri M Dalvi, Tanya Benson, Sree Lakshmi, Tammy Ho Wye Yann, Trijanya Gowda

**Affiliations:** a Professor, Department of Public Health Dentistry, Bapuji Dental College and Hospital, Davangere, Karnataka, India. Concept, design of the study, manuscript editing and definition of intellectual content.; b Postgraduate Student, Department of Public Health Dentistry, Bapuji Dental College and Hospital, Davangere, Karnataka, India. Manuscript preparation, manuscript editing and statistical analysis.; c Undergraduate Student, Bapuji Dental College and Hospital, Davangere, Karnataka, India. Literature search and data acquisition.; d Undergraduate Student, Bapuji Dental College and Hospital, Davangere, Karnataka, India. Literature search and data acquisition.; e Undergraduate Student, Bapuji Dental College and Hospital, Davangere, Karnataka, India. Literature search and data acquisition.; f Undergraduate Student, Bapuji Dental College and Hospital, Davangere, Karnataka, India. Literature search and data acquisition.

**Keywords:** nurses, oral health, oral hygiene

## Abstract

**Purpose:**

Nurses are the professional group who most often provide care. Studies have shown that oral healthcare is being neglected by general healthcare professionals, including nurses. With proper health-related behaviour, knowledge and attitude, they can play an important role in health education and act as a role model for patients. The aim of the study was to assess knowledge, attitude and practices related to oral health among nursing (Bsc) students in Davangere city, Karnataka, India.

**Materials and Methods:**

A cross-sectional survey was carried out among 365 BSc Nursing students at their college premises in Davangere city. Data was collected using 25 items self-administered questionnaire which was validated. One-way analysis of variance (ANOVA) and Student’s unpaired t tests were used considering p ≤0.05 as statistically significant.

**Results:**

The mean knowledge scores of final and third year nursing students were significantly higher than the first and second year students (p = 0.01). The majority of the students felt the need for regular visits to dentist (72.6%) and felt that oral healthcare was an important part of nursing care (91.2%). They also felt the need to collaborate with dentists (78.1%). The majority of them brushed their teeth twice daily (74.2%) and had the habit of mouth rinsing (67.4%) and referred their patients to dentists (61.4%).

**Conclusion:**

The knowledge related to oral health among nursing students was good. The majority believed that oral healthcare was an important part of nursing care, hence the training of nursing students pertaining to oral health education and upgrade of their academic curriculum should be prioritised.

Oral health is defined by the World Health Organization (WHO) as a state of being free from chronic mouth and facial pain, oral and throat cancer, oral sores, birth defects such as cleft lip and palate, periodontal (gum) disease, tooth decay and tooth loss, and other diseases and disorders that affect the oral cavity.^16^ It is evident that oral health is an important component of general health; it is the mirror of our general health. Several studies have shown that oral healthcare is being neglected by general healthcare professionals, including nurses.^1,2,4,7,8,9,14^ Elderly people and infants who are considered as care dependents are increasing in number day by day.

Nurses are the professional group who most often provide these groups care. One study that examined nursing home residents found that 78% needed help with oral hygiene, but only 7% received such help from nurses.^6^ This is due to low priority, high workload, lack of training and poor understanding of the importance of oral health among nurses. Nurses are specialised in nursing care, preventive information and health promotion and it is important that their own oral healthcare knowledge is good, and that their oral health behaviour and attitude conform to professional recommendations. With proper health-related behaviour, knowledge and attitude, they can play an important role in health education and act as a role model for patients. Moreover, it has been indicated that oral healthcare has not been a sufficient part of the training of nursing students and that the literature used was outdated, resulting in oral healthcare not being carried out by nurses on the basis of the newest available evidence.^1,14,15^ Therefore, we should emphasise the importance of maintaining good oral health among nurses.

Hence a cross-sectional survey was planned to assess the knowledge, attitude and practices related to oral health among nursing students in Davangere city, Karnataka, India. This would thus create a baseline data to plan oral health educational models to train the nurse population to provide proper oral health education and guidance in maintaining good oral health.

## Materials and Methods

The present study was a descriptive, cross-sectional questionnaire survey conducted among first, second, third year and final year students of BSc Nursing from five nursing institutes /colleges in Davangere city, Karnataka, India. The data was collected from the nursing students at their respective college premises. All the first, second, third and final year BSc Nursing students (400 students) from the following colleges/institutes who consented to participate formed the study sample (whole sample).

1. Raghavendra Nursing College, SS Layout (n = 72)
2. Kumuda Institute of Nursing, SS Layout (n = 44)
3. Bapuji College of Nursing, MCC B block (n = 200)
4. Sanjeevni Nursing College, Anjanaya Badavane (n = 53)
5. Mritunjaya School of Nursing, PJ Extenison (n = 31)

Eligibility criteria:

- Nursing students who voluntarily consented to participate.
- Nursing students studying in first, second, third and final year BSc Nursing course.

Ethical approval was obtained from the Institutional Review Board of Bapuji Dental College and Hospital, Davangere. Permission was obtained from the Heads of the concerned institutions to conduct the survey. Voluntary written informed consent was obtained from the study participants after explaining them about the purpose of conducting the study and procedure of collecting the data through questionnaire.

### Questionnaire Details

Data was collected using pretested, validated, self-administered questionnaire containing two sections. The questionnaire was designed in English language. Section one had provision to record demographic details (name, age, gender and college address). Section two included questions to assess the knowledge, attitude and practices related to oral health among the nursing students.

Considering the three main constructs (knowledge, attitude and practices), 25 items were framed. The first 15 items were designed to assess the knowledge of the participants regarding oral health. Responses for item numbers 1–13 were placed on three-point Likert scale, and item numbers 14–15 were multiple-choice questions. The responses for items assessing knowledge were assigned scores. The scores for all items assessing knowledge were summed up which represented the knowledge score of the participant and mean knowledge score was calculated. Five items (item numbers 16–20) were designed to assess the attitudes of the participants regarding oral health the responses of which are on a three-point Likert scale and five multiple-choice questions (item numbers 21–25 ) were designed to assess the practices related to oral health.

### Validity of the Questionnaire

Content validity of the questionnaire was assessed by five experts: dental nurses (2), public health dentists (2), final year BSc Nursing students (1). The questionnaire was assessed for relevance, simplicity, clarity and ambiguity. Content validity index for relevance was 0.76, clarity, simplicity and ambiguity were 0.68, 0.82 and 0.72 respectively. All the components had a CVI score more than 0.6 hence the questionnaire was validated. Suggestions provided by experts to improve the content validity was considered and the questionnaire was reframed.

### Method of Data Collection

The questionnaire was distributed to the nursing students in the respective departments where they were posted after explaining about the purpose of the survey. The questionnaire was distributed to them by investigators. Participants were instructed not to discuss any answers with their friends. They were also instructed to approach an investigator if they had any doubts pertaining to the questionnaire. Participants were instructed to fill in the questionnaire and return it to the investigator within 30 min.

### Statistical Analyses

The data obtained was compiled systematically in Microsoft Excel and subjected to statistical analysis using Statistical Package for Social Sciences software (SPSS version 21.0, IBM, NY, USA). Data (responses) pertaining to attitude and practices was represented in percentages. Data pertaining to knowledge was continuous data and for comparison of knowledge scores between two independent groups were performed using Student’s unpaired t test and for comparison of knowledge scores between more than two independent groups was done using one-way ANOVA test.

## Results

A total of 365 out of 400 students responded to the survey. The overall response rate was 91%. The average age of study participants was 20 years. The majority of nursing students were females (85.5%). First and second year BSc Nursing students constituted 65% of the total participants, whereas third and final year students constituted 35%. The responses for items assessing knowledge were assigned scores. The scores for all items assessing knowledge were summed up which represented the knowledge score of the participant and mean knowledge score was calculated. Criteria for differentiating into good, average and poor knowledge was decided by totalling the response codes for each item related to assessment of knowledge. The total of the response codes was set at 0–5.6 for poor, between 5.7 and 11 for average and 11–17 onwards for good. Knowledge related to oral health was good among study subjects since their mean knowledge score was 13.09. The mean knowledge scores of final year and third year students were significantly higher than the first and second year students (p = 0.01) (Table 1). There was no statistically significant difference (p = 0.413) between the mean knowledge scores of males (11.23) and females (13.08).

**Table 1:** Distribution of mean knowledge scores between different year BSc Nursing students

Si No	Year of study	Mean knowledge scores	ANOVA test
	First	9.08	f value = 3.490 p value = 0.012*
	Second	9.84	
	Third	11.54	
	Final	14.06	
	Total	13.08	

* indicates statistically significant difference for p ≤0.05.

Majority of students (more than 70%) felt that oral health was related to general health, by proper toothbrushing and flossing, dental decay and gum disease could be prevented and frequent consumption of sticky sweets caused tooth decay. Over 50% of them felt alignment of teeth was done for aesthetic and functional purpose, para-functional habits like mouth breathing, thumb sucking and nail-biting among children could have an effect on jaw and facial growth, Mouth guards prevented sports-related injuries to oral structures, loss of teeth in old age could be prevented by proper maintenance of oral health and soft drinks caused weakening of teeth and tobacco consumption was the main cause for oral cancer (Table 2). Over 30% of nursing students did not know that gum disease is linked with diabetes, ill-fitting dentures, preterm delivery, sharp tooth and cardiac diseases (Table 2).

**Table 2:** Distribution of response rates to questions related to oral health knowledge

Si no	Question	Agree (%)	Disagree (%)		Don’t know (%)
	Oral health is related to general health	323 (88.5%)	36 (9.9%)		6 (1.6%)
	Proper maintenance of milk teeth is as important as permanent teeth	165 (45.2%)	177 (48.2%)		23 (6.3%)
	Dental decay and gum disease are caused by plaque (tartar)	156 (42.7%)	108 (29.6%)		101 (27.7%)
	By proper tooth brushing and flossing dental decay and gum disease can be prevented	299 (81.9%)	46 (12.6%)		20 (5.5%)
	Fluoride prevents against tooth decay	171 (46.8%)	143 (39.2%)		51 (14%)
	Germs that cause tooth decay are transmitted from mother to her child	122 (33.4%)	227 (62.2%)		16 (4.4%)
	Frequent consumption of sticky sweets causes tooth decay	293 (80.3%)	54 (14.8%)		18 (4.9%)
	Alignment of teeth is done for aesthetic and functional purpose	185 (50.7%)	80 (21.9%)		100 (27.4%)
	Para-functional habits like mouth breathing, thumb sucking and nail-biting among children can have an effect on jaw and facial growth?	243 (66.6%)	97 (26.6%)		25 (6.8%)
	Mouth guards prevent sports-related injuries to oral structures	248 (67.9%)	78 (21.4%)		39 (10.7%)
	A tooth avulsed (come out of the socket) due to trauma can be replaced into the socket	152 (41.6%)	102 (27.9%)		111 (30.4%)
	Loss of teeth in old age can be prevented by proper maintenance of oral health	247 (67.7%)	102 (27.9%)		16 (4.4%)
	Soft drinks cause weakening of teeth	247 (67.7%)	98 (26.8%)		17 (4.7%)
	Following causes oral cancer	Tobacco		222 (60.8%)	
		Pan-chewing		54 (14.8%)	
		Ill-fitting prosthesis		3 (0.8%)	
		Sharp teeth		5 (1.4%)	
		All of the above		70 (19.2%)	
		Don’t know		11 (3.0%%)	
	Gum disease is linked with	Diabetes		109 (29.9%)	
		Pregnancy		27 (7.4%)	
		Preterm birth weight		30 (8.2%)	
		Cardiac diseases		25 (6.8%)	
		All of the above		51 (14%)	
		Don’t know		123 (33.7%)	

Over 70% of the students felt the need for regular visits to dentist (72.6%), felt that oral healthcare was important part of nursing care (91.2), felt the need to collaborate with dentists (78.1%) and felt oral health-related subjects should be updated and expanded in the nursing education (82 %) (Table 3). Majority of them brushed their teeth twice daily (74.2%) with toothbrush and toothpaste (94.2%), had the habit of mouth rinsing (67.4%) and referred their patients to dentists (61.4%) (Table 4).

**Table 3:** Distribution of response rates to questions related to attitude towards oral health

Si no	Question	Yes (%)	No (%)	Don’t know
	Do you feel regular visit to dentist for oral health check-up is necessary?	265 (72.6%)	91 (24.9%)	9 (2.4%)
	Do you think oral healthcare can be an important part of nursing care?	333 (91.2%)	21 (5.8%)	11 (3.0%)
	Do you feel oral care is given less emphasis in your curriculum?	121 (33.2%)	166 (45.5%)	78 (21.4%)
	Do you feel oral health should be updated and expanded in the nursing education?	300 (82.2%)	36 (9.9%)	29 (7.9%)
	Do you feel the need to collaborate with dentists in order to provide oral healthcare for patients?	285 (78.1%)	36 (9.9%)	44 (12.1%)

**Table 4:** Distribution of response rates to questions related to practices towards oral health

Si no	Question	Options	Response
	How often do you visit your dentist?	Once in 6 months	148 (40.5%)
		Once in a year	33 (9%)
		Whenever there is a problem	161 (44.1%)
		Never visited	23 (6.3%)
	Do you refer your patients to the dentist when necessary?	Yes	224 (61.4%)
		No	46 (12.6%)
		Sometimes	95 (26.2%)
	How many times you brush your teeth in a day?	Once	82 (22.5%)
		Twice	271 (74.2%)
		More than twice	12 (3.3%)
	What do you use to clean your teeth?	Toothbrush and toothpaste	344 (94.2%)
		Toothbrush and tooth powder	14 (3.8%)
		Any other	7 (1.9%)
	Do you have the habit of mouth rinsing?	Yes	246 (67.4%)
		No	74 (20.3%)
		Sometimes	45 (12.3%)

## Discussion

This study was conducted to know the knowledge, attitude and practices related to oral health among BSc Nursing students in Davangere city, Karnataka, India. Davangere city was selected as it was considered as the educational hub in Karnataka consisting of many renowned institutes related to medical, dental and nursing disciplines. As per 2011 census, Davangere city had a population of 434,971. Males constituted of 52% and females 48% of the population. It had a literacy rate of 85%, higher than the national average of 70.04% out of which male literacy rate was 89% and female literacy rate was 81%.^5^

The knowledge related to oral health was good among nursing students. These results are in accordance with study results of Bhattarai et al and Alsrour et al.^2,4^ However in a study conducted by Grønkjær et al, nursing students had good oral health knowledge regarding plaque and dental caries.^7^ The knowledge about periodontal diseases such as gingivitis and periodontitis was insufficient. Few studies have shown that oral health knowledge was expected to be good among dental students as compared to medical and paramedical students like nursing students.^3,9,12^ In a study done by Smadi et al, the oral health-related knowledge was poor among nursing students.^13^

In this study, a majority of the students considered oral health as an important part of nursing care and important for general health. These results are in accordance with other studies which examined attitudes regarding oral health among nurses.^7,8,13^ This positive attitude towards oral health was reflected in the majority of students, ie, of wanting oral health training to be updated and expanded in the nursing education. Several studies have reported that nurses have high levels of interest in updating themselves on oral health and care.^7,8^ However, little time is devoted to oral health and disease topics in the training of non-oral health professionals such as nursing students. By providing oral health education programmes, more nursing students and nurses can improve the oral health of patients in their care. Nurses make up the largest proportion of healthcare professionals and encounter far more patients who need oral care than other healthcare professionals. Therefore, it is essential that nurses have sufficient knowledge about oral health in order that they may make appropriate referrals and interventions. Collaboration between oral healthcare professionals and general healthcare professionals could raise awareness of the importance of oral health for the general health. In addition, educational institutions should play an active role in creating interdisciplinary opportunities where dental, medical and nursing students may develop knowledge about oral health.

However, a study by Khairnar et al reflected a poor attitude of nursing students towards oral health compared to other healthcare professionals due to poor knowledge about oral healthcare and poor oral hygiene habits.^9^

This study revealed that the majority of nursing students were using toothbrush and toothpaste to clean their teeth, twice daily. These findings are in accordance with the results of Grønkjær et al and Kaira et al.^7,8^ However, the reason most often given to visit the dentist was mainly because of dental problems, which reflects less importance given by nurses towards regular dental check-ups. This finding is in accordance with a study done by Kaira et al in Rohilkhand, India.^8^ This may be due to the long and unpredictable working hours of nurses which may not facilitate them to make for regular oral health check-ups for themselves in spite of them realising the importance of regular health check-ups towards oral health maintenance.

The majority of respondents in this study were female and perhaps women tend to be more positive about oral health and care than men.^10,11^ Since it also includes nursing students just from from Davangere city, the results cannot be generalised to other populations. The use of self-administered questionnaires limits the conclusion. Some of the questions required the students to recall past events, which might have caused memory bias. The questionnaire did not contain items to assess the quality of received training regarding provision of oral health for patients. Also, no items asked the participants to self-evaluate their knowledge and skills regarding provision of oral health for patients. The results of the study may motivate further studies within this field and may be helpful in planning oral health training, targeting nursing students or other healthcare professionals. The further training of nursing students pertaining to oral health education and upgrade of their academic curriculum related to oral health is recommended.

## Conclusion

From the present study we concluded that knowledge related to oral health was good among this study’s subjects. The majority of the students felt the need for regular visits to dentist and felt that oral healthcare was an important part of nursing care. Most brushed their teeth twice daily and had the habit of mouth rinsing and referred their patients to dentists. They also felt the need to collaborate with dentists for providing optimum oral care for the patients.
